# CCL20 promotes lung adenocarcinoma progression by driving epithelial-mesenchymal transition

**DOI:** 10.7150/ijbs.73275

**Published:** 2022-06-27

**Authors:** Tao Fan, Shuofeng Li, Chu Xiao, He Tian, Yujia Zheng, Yu Liu, Chunxiang Li, Jie He

**Affiliations:** 1 Department of Thoracic Surgery, National Cancer Center/National Clinical Research Center for Cancer/Cancer Hospital, Chinese Academy of Medical Sciences and Peking Union Medical College, Beijing, 100021, China.; 2 Department of Oncology, Renmin Hospital of Wuhan University, 238th Jiefang Road, Wuhan 430060, China.; 3 Department of Colorectal Surgery, National Cancer Center/National Clinical Research Center for Cancer/Cancer Hospital, Chinese Academy of Medical Sciences and Peking Union Medical College, Beijing, China.; 4 Department of Interventional Therapy, National Cancer Center/National Clinical Research Center for Cancer/Cancer Hospital, Chinese Academy of Medical Sciences and Peking Union Medical College, Beijing, China.

**Keywords:** CCL20, EMT, immune checkpoint blockade, tumor immunology

## Abstract

C-C motif chemokine ligand 20 (CCL20) participates in multiple oncogenic processes, but its role in lung adenocarcinoma (LUAD) is unclear. Herein, we explored the mechanism by which CCL20 works in LUAD progression. We performed bioinformatical analyses based on the complete transcriptome sequencing data from 1544 LUAD cases in 4 independent cohorts to evaluate signaling pathways regulated by CCL20. We established A549 and H358 cell lines with CCL20 knockdown to explore how CCL20 promotes tumor progression *in vitro* and *in vivo* experiments. Using another independent cohort of 348 urothelial carcinoma patients treated with the anti-PD-L1 agent (atezolizumab), we explored the synergistic effect of CCL20 and TGF-β on immunotherapy efficacy. High CCL20 expression is a poor prognostic marker for LUAD patients, and is associated with enhanced epithelial-mesenchymal transition (EMT), inflammatory response, and activated TNF pathway in LUAD. CCL20 knockdown restrained the EMT process and cell proliferation of LUAD cells *in vitro* and *in vivo*. Low CCL20 expression blocked the detrimental effects of high TGF-β on survival and effectively improved patients' response to anti-PD-L1 therapy. Collectively, we revealed the underlying mechanisms by which CCL20 promotes LUAD progression based on the largest sample size. The synergistic inhibitory effect of CCL20 and TGF-β on immune-checkpoint blockade therapy efficacy provides new views of immunotherapy resistance.

## Introduction

Lung cancer is the leading cause of cancer-associated death. According to the latest statistic data, lung cancer caused 2,206,771 new cases (account for 11.4% of all cancer diagnoses) and 1,796,144 new deaths (account for 18.0% of overall cancer mortality) in 2020 1. Lung adenocarcinoma (LUAD) has become the most prevalent pathological type, making up approximately >50% of all lung cancer cases 2.

Chemokines are categorized into C-motif (C), C-C motif (CC), C-X-C motif (CXC), and CX3C subfamilies according to the difference in the position of Cys residues within the N-terminal 3. They extensively participate in malignant processes through their complicated regulation of cancer cells and non-cancer cells, and their interaction with other cytokines within the tumor microenvironment (TME) 4. CCL20, also known as macrophage inflammatory protein (MIP)-3α, Exodus-1, and liver and activation-regulated chemokine (LARC) 3, has exhibited its oncogenic effects in various tumors. Many studies defined CCL20 and its specific receptor CCR6 as prognostic markers and potential interventional targets, including colorectal cancer 5, breast cancer 6, and prostate cancer 7. Mechanistically, CCL20 produced by cancer cells can render the cell itself to a more malignant phenotype. For example, CCL20 promoted the secretion of MMP2/9 and cell invasion ability in the basal-like triple-negative breast cancer cell lines, and treatment of anti-CCL20 antibodies could block the osteolytic breast cancer bone metastasis in mice 8. Moreover, CCL20 and CCR6 comprise a chemokine regulatory axis to regulate the behavior of cancer cells and immune cells concurrently, thereby shaping tumor immune microenvironment (TIME) states and influencing the combat between the immune system and the tumor. This regulatory effect has been reported in ovarian cancer and hepatoma 9, 10. However, the characteristics of CCL20 in LUAD have not been clarified.

Our previous study established a five-chemokine and chemokine receptor-based signature as clinical prognosis and immunotherapy efficacy predictors of LUAD 11. As one part of the predictive signature, CCL20 was identified to play a critical role in LUAD by immune and non-immune related mechanisms. Therefore, we further explored the molecular details behind the impacts of CCL20 on LUAD progression and CCL20-associated opportunities to improve immunotherapy efficacy.

In this study, we performed bioinformatics analysis, *in vitro* experiments, and established animal models to confirm the tumor-promoting effects of CCL20 on LUAD. We found that CCL20 promotes LUAD progression mainly via the EMT pathway, and TGF-β plays a synergetic role in this process. Furthermore, the synergetic effects of CCL20 and TGF-β led to a poor response of patients to immune checkpoint blockade (ICB) therapy.

## Results

### High expression of CCL20 leads to a poor prognosis in patients with LUAD

To thoroughly survey the landscape of CCL20 expression in cancer, we compared the expression level of CCL20 in tumor tissues and tumor-adjacent tissues of pan-cancer using the RNA-seq data from TCGA pan-cancer datasets. We found that CCL20 expression was generally upregulated in tumor tissues of most cancer types compared with tumor-adjacent tissues. In some cancers including LUAD and LUSC, the differential expression levels had statistical significance (Figure S1).

**Figure 1:**
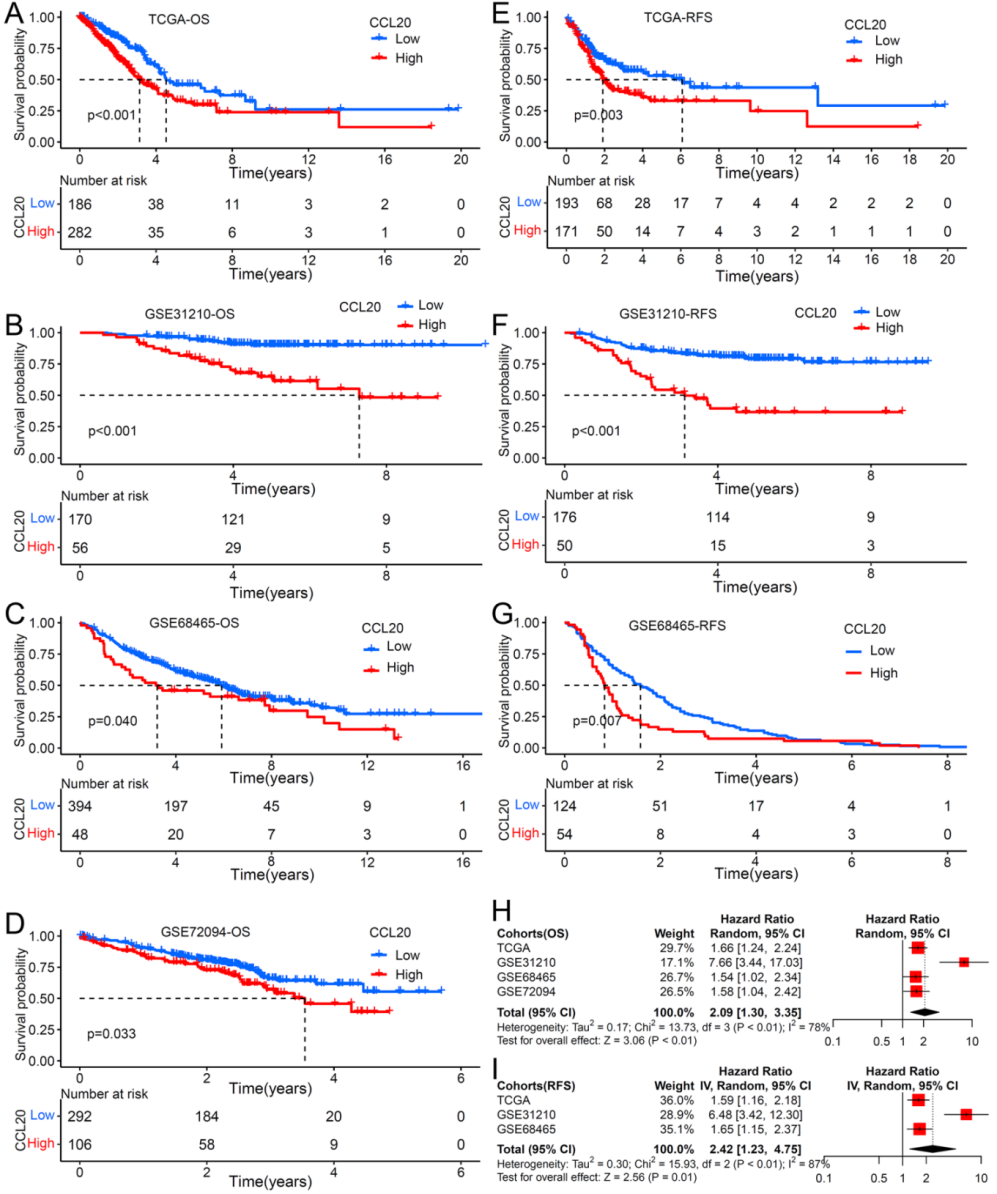
The impact of CCL20 expression on prognosis of patients with LUAD. Overall survival (OS) analysis for patients with low or high CCL20 level in four independent cohorts: (A) TCGA (n=477); (B) GSE31210 (n=226); (C) GSE68465 (n=442); (D) GSE72094 (n=398). Relapse free survival (RFS) analysis for patients with low or high CCL20 level in three independent cohorts: (E) TCGA (n=477); (F) GSE31210 (n=226); (G) GSE68465 (n=178). (H) A meta-analysis for OS related cohorts. (I) A meta-analysis for RFS related cohorts.

Previous studies reported that the CCL20 expression level in tumor tissues is associated with clinical prognosis in multiple cancer types 12, but its prognosis value in LUAD is unknown. We further investigated whether CCL20 expression in tumor tissues has effects on clinical outcomes of LUAD patients. We performed survival analysis using TCGA LUAD cohorts and found that patients with higher CCL20 expression levels had poorer RFS and OS than patients with low CCL20 expression levels (Figure 1A, 1E). We validated the prognostic relevance using the other three GEO cohorts, including GSE31210, GSE68465, and GSE72094, and came to the same conclusion (Figure 1B-D, 1F-1G), that was the higher CCL20 expressed, the poorer OS and RFS showed. The demographics of TCGA and GEO cohorts were listed in Table 1. The meta-analysis result for OS was significant in all 4 cohorts (combined HR = 2.09, 95%CI= 1.30-3.35, meta-analysis P< 0.01). TCGA, GSE31210, and GSE684653 cohorts had statistic significances for RFS in meta-analysis (combined HR = 2.42, 95%CI= 1.23-4.75, meta-analysis P<0.01). Univariable and multivariable Cox regression analysis based on TCGA cohorts showed that CCL20 expression still had prognostic ability after excluding the possible confounding factors including ages, genders, TNM stages, and smoking history (Table 2). Collectively, the CCL20 expression level in tumor tissues had a predictive prognosis for LUAD. High CCL20 levels were independently associated with survival.

### Biological pathways of related to CCL20

**Figure 2:**
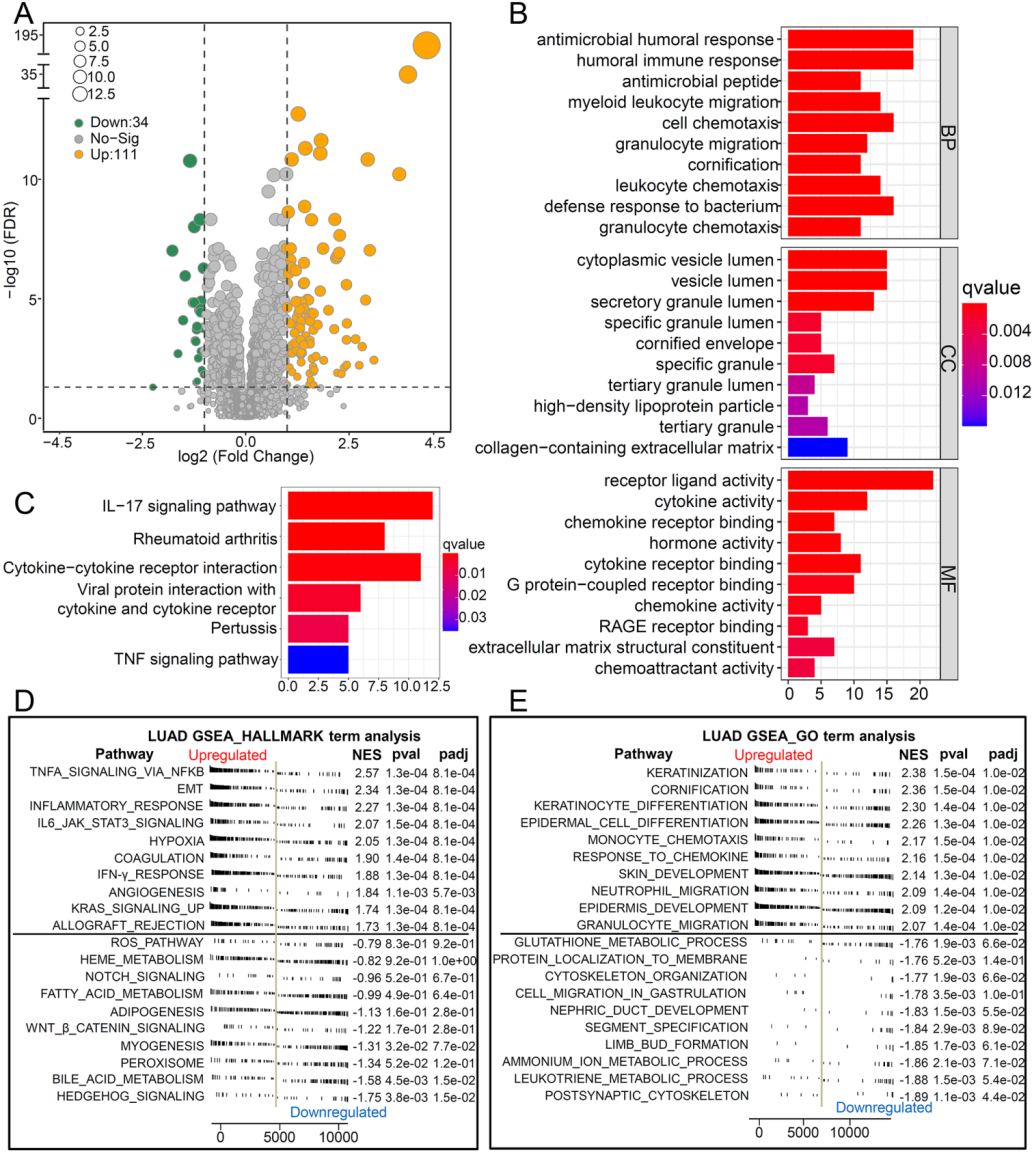
Biological pathways of related to CCL20. (A) The volcano map showed the 145 DEGs between patients with high CCL20 levels and patients with low CCL20 levels. GO (B) and KEGG (C) enrichment analysis for these identified DEGs. GSEA hallmark term analysis (D) and GO term analysis (E) for high and low CCL20 expression groups.

To explore the biological pathways by which CCL20 influences LUAD progression, we performed DEG analysis and functional enrichment analysis. We found 111 upregulated and 34 downregulated genes between the high and low CCL20 expression subgroup from TCGA cohorts of 468 patients (Figure 2A). Furthermore, the GO analysis of the 145 DEGs demonstrated that these DEGs mainly mapped to infection immunity, leukocyte migration, cell chemotaxis, and cytokine activity (Figure 2B). Meanwhile, KEGG analysis revealed that these genes were involved in IL-17 and TNF signaling pathways (Figure 2C). We performed a GSEA analysis to determine CCL20 expression-related biological pathways further (Figure 2D-E). The GSEA hallmark term analysis showed that the TNFA_SIGNALING_VIA_NFKB, EMT, and INFLAMMATORY_RESPONSE were the most significantly enriched pathways in high CCL20 groups. Additionally, GO-term analysis showed that KERATINIZATION and CORNIFICATION were upregulated in high CCL20 expression groups. Taken together, CCL20 is extensively associated with tumor immunity, cytokine activity, inflammation, and oncogenic signaling pathways in LUAD.

### Patients with low CCL20 expression showed specific characteristics of programmed cell death

**Figure 3:**
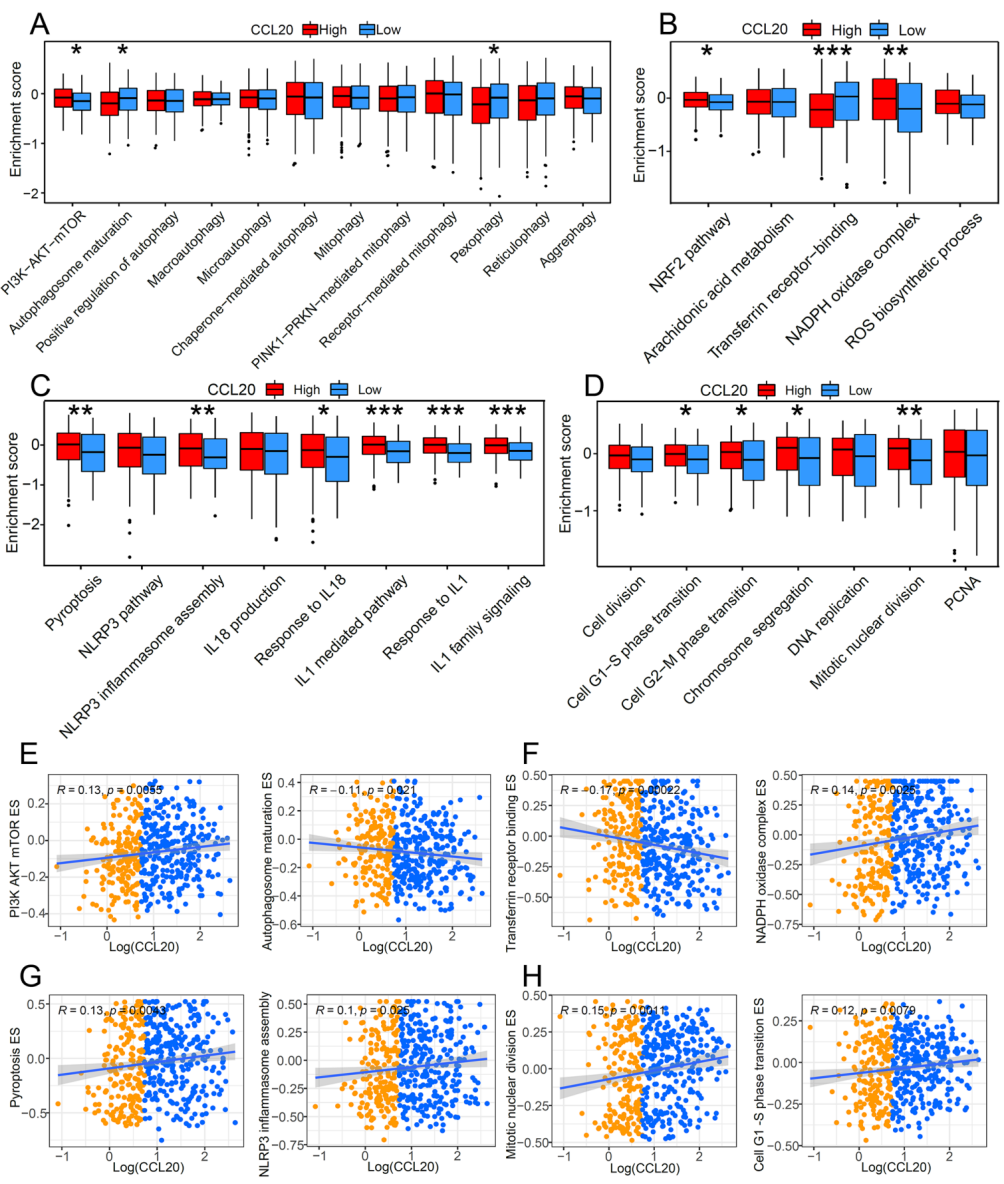
The impact of CCL20 on programmed cell death and cell proliferation. Comparison of autophagy-related pathway enrichment scores (A), ferroptosis-related pathway enrichment scores (B), and pyroptosis-related pathway enrichment scores (C) between patients with high expression of CCL20 and patients with low expression. (D) Differences in cell proliferation pathways between high and low CCL20 expression groups. (E) Correlation analysis of CCL20 and major autophagy-related pathways. (F) Correlation analysis of CCL20 and major ferroptosis-related pathways. (G) Correlation analysis of CCL20 and major pyroptosis-related pathways. (G) Correlation analysis of CCL20 and major cell proliferation pathways.

Programmed cell death is a critical mechanism to maintain organic homeostasis by eliminating dysfunctional or malignant transformed cells to block malignancy. Suppressed programmed cell deaths and activated cell proliferation are hallmarks of cancer development. Previous studies found that chemokines can promote the apoptosis resistance of cancer cells, leading to malignant transformation and cancer progression 13. However, the correlation between CCL20 and programmed cell death in cancer is unclear, so we further investigated whether CCL20 expression affects cell death and cell proliferation. GSVA analysis showed a significant difference in the enrichment scores (ESs) of autophagy, pyroptosis, and ferroptosis-related pathways between the CCL20 high and CCL20 low groups (Figure 3A-D). In the CCL20-high group, the ESs of inhibitory markers for autophagy and ferroptosis were lower than CCL20-low groups (Figure 3A-B). In contrast, pyroptosis and cell proliferation-related markers are generally upregulated in the CCL20-high group (Figure 3C-D). We further analyzed the correlation between CCL20 expression and critical markers and components ESs of these pathways (Figure 3E-H). AKT/mTOR signaling pathway inhibits autophagy activation, and the positive correlation between CCL20 and PI3K, AKT, and mTOR ESs indicated that the repressive role of CCL20 in autophagy (Figure 3E). Transferrin receptor is a marker of cells undergoing ferroptosis 14, and NADPH is a negative marker of ferroptosis. The scatter diagram suggested that CCL20 is associated with downregulated ferroptosis signaling (Figure 3F). NLRP3 is a critical component of inflammasome for pyroptosis initiation. The positive correlation between CCL20 levels and NLRP3 ES shown in Figure 3G indicated that CCL20 promoted pyroptosis. Meanwhile, CCL20 promoted cell proliferation (Figure 3H). These findings suggested that CCL20 extensively regulates cell deaths and cell proliferation, and the dysregulation of cell deaths may be a resource for the tumor-promoting role of CCL20 in LUAD.

### The relationship between CCL20 and three tumorigenic pathways

**Figure 4:**
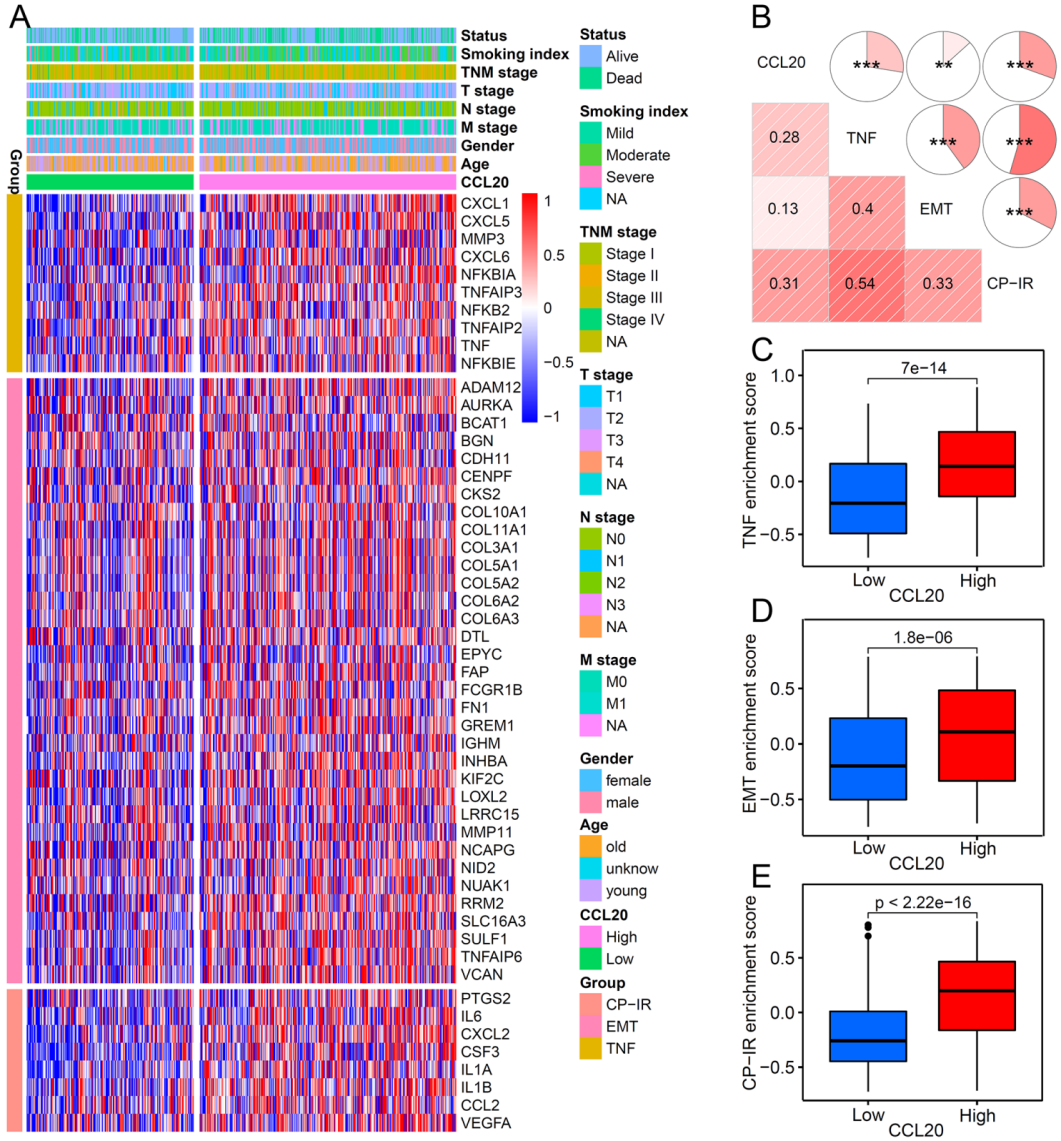
The relationship between CCL20 and three tumorigenic pathways. (A) Heatmap was used to visualize TNF, EMT, and CP-IR-related gene expression profiles. (B) Correlogram was generated based on Pearson r-value between CCL20 expression and the three tumorigenic pathways. (C) Differences in TNF pathway between high CCL20 and low CCL20 groups. (D) Differences in EMT between high CCL20 and low CCL20 groups. (E) Differences in CP-IR signature between high CCL20 and low CCL20 groups.

To further validate the correlation between CCL20 and EMT, TNF, and inflammatory signaling pathways in LUAD presented in GO, KEGG, and GSEA analysis (Figure 2B-E), we investigated the expression of critical genes of these signaling pathways in high and low CCL20 expression subgroups from TCGA cohort. The heatmap showed that EMT, TNF, and CP-IR 15 related gene profiles were generally upregulated in the CCL20-high subgroup (Figure 4A). GSVA analysis and correlogram showed a significant difference in the activation of these pathways between CCL20 high and CCL20 low groups (Figure 4B-E). Moreover, we analyzed the association between EMT-related pathways and CCL20 expression levels in LUAD based on TCGA and GSE31210 cohorts. We found that CCL20 was involved in multiple EMT-related pathways, including RHO GTPases, HIF1 pathway, RHO GTPases effectors, RHO GTPases activate formins, and RHO GTPases activate IQGAPs in all two cohorts (Figure S2A-D). TGF-β1 is a crucial inducer of EMT, promoting the EMT process through transcriptional and post-transcriptional regulation of numerous cancer-related transcriptional factors and growth factors 16, 17. Notably, CCL20 expression was markedly correlated with the TGF-β1 pathway in two cohorts. To make sure whether there are coordinated prognostic values of CCL20 and TGF-β1 for LUAD, we stratified the patients from TCGA and GSE31210 cohorts into 4 subgroups according to the expression levels of CCL20 and TGF-β1 and then performed survival analysis for each subgroup. We found that patients with high CCL20 and high TGF-β1 expression had the poorest OS and RFS, and patients with low CCL20 and low TGF-β1 expression had the best OS and RFS (Figure S2E-F). Briefly, CCL20 promoted LUAD progression via the EMT pathway mediated by TGF-β1.

### Inhibition of CCL20 expression can significantly promote lung adenocarcinoma cell apoptosis and inhibit cell proliferation and EMT

**Figure 5:**
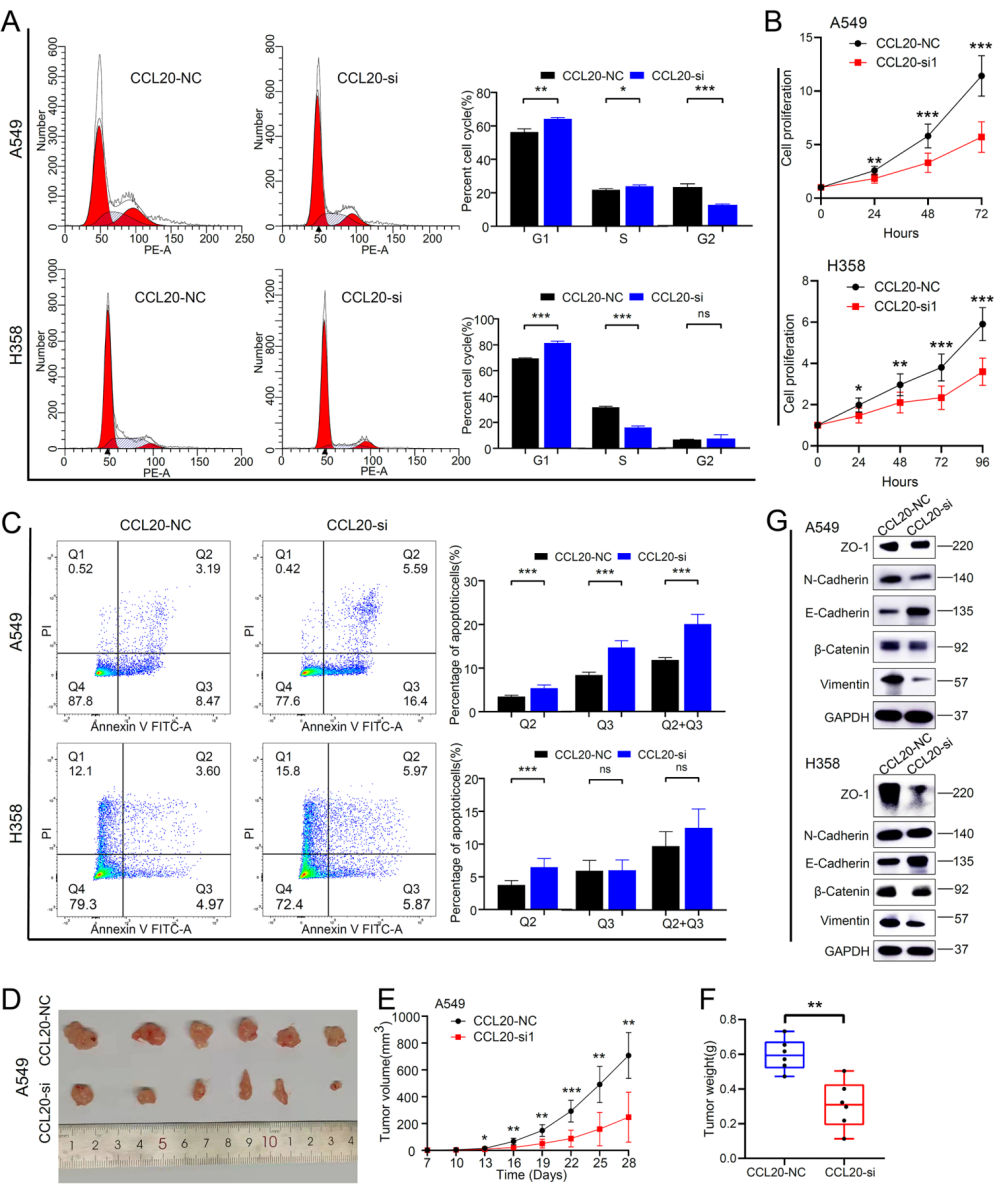
CCL20 knockdown suppressed tumor malignant phenotype and inhibited pathological Epithelial-Mesenchymal Transition. (A) Cell cycle analysis in CCL20 knockdown A549 and H358 cell lines and control cell lines. (B) Cell proliferation analysis in CCL20 knockdown A549 and H358 cell lines and control cell lines using CCK8 assay. (C) Flow cytometry was used to detect the level of cisplatin-induced apoptosis in CCL20 knockdown A549 and H358 cell lines and control cell lines. (D-F) Subcutaneous tumors developed from CCL20 knockdown A549 cells and control cells. (G) Western blot was used to evaluate the expression levels of the EMT-related markers in CCL20 knockdown A549 and H358 cell lines and control cell lines.

We performed a series of *in vitro* and *in vivo* experiments to validate our findings from the bioinformatic analysis. The CCL20 siRNA knockdown efficiency in A549 and H358 cell lines was detected by western blot (Figure S3). We found that more A549 and H358 cells stalled at the G1 phase after CCL20 knockdown in the cell cycle array (Figure 5A). The difference in the percentage of A549 cells in G1, S, and G2 are statistically significant between knockdown and control groups.

The percentage of H358 cells in the G2 phase showed no difference between knockdown and control groups (Figure 5A). Cell proliferation assays demonstrated that the proliferation rates of A549 and H358 cells with CCL20 knockdown were lower than control groups (Figure 5B). Given the finding that CCL20 may be indicated in regulating cell deaths, we did cell apoptosis detection and found that CCL20 knockdown significantly promoted apoptosis (Figure 5C). We had screened out that EMT is likely to be the predominant mechanism by which CCL20 accelerates LUAD progression, so we performed a western blot analysis to validate whether CCL20 affects the expression of EMT-related proteins. The protein levels of N-Cadherin and β-Cadherin decreased, whereas E-cadherin and Vimentin increased in the two cell lines after CCL20 knockdown, certifying CCL20 promoted the EMT process of LUAD cells (Figure 5G). We further performed subcutaneous tumorigenesis experiments, exploring whether CCL20 keeps the tumor-promoting effects within complicated intracorporeal environments. The result showed that CCL20 knockdown significantly repressed tumor growth in the xenograft model (Figure 5D-F). Taken together, we confirmed that CCL20 promotes LUAD progression and the activation of the EMT process is a critical mechanism.

### CCL20 and TGF-β had a synergistic effect on cancer immunotherapy

**Figure 6:**
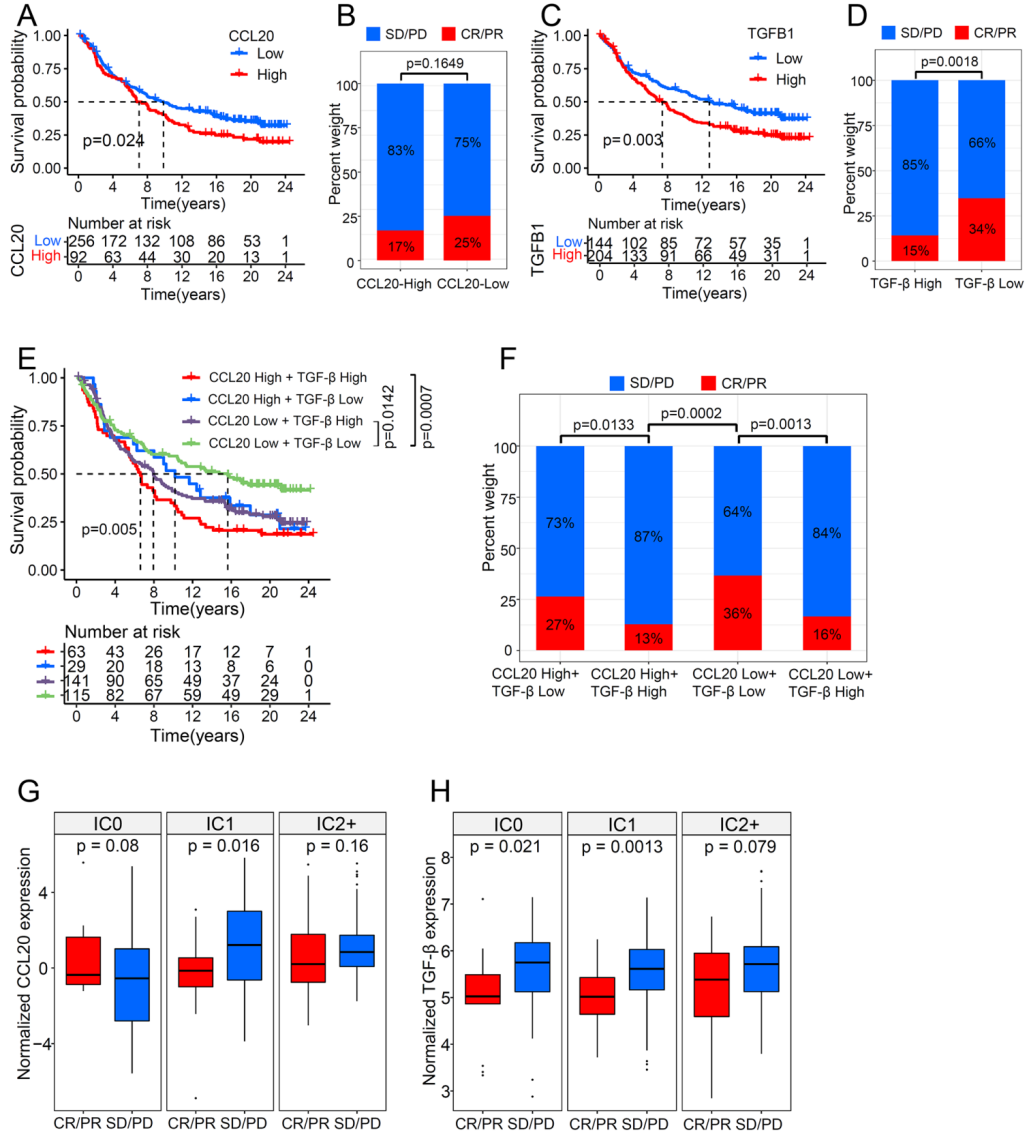
CCL20 and TGF-β had a synergistic effect on urothelial cancer immunotherapy. (A) Urothelial cancer patients with low expression of CCL20 had a significant survival advantage. (B) Urothelial cancer patients with high CCL20 expression were more likely to not respond to PD-L1 blockade. (C) Urothelial cancer patients with low expression of TGF-β had a significant survival advantage. (D) TGF-β expression was significantly associated with non-response to PD-L1 blockade. (E) CCL20 and TGF-β had a synergistic effect on reduced overall survival of urothelial cancer patients treated with PD-L1 blockade. (F) High expression of CCL20 and TGF-β was significantly associated with non-response to PD-L1 blockade. (G) CCL20 was significantly associated with PD-L1 blockade response in the IC1 phenotype (two-tailed t-test p = 0.016). (H) TGF-β was significantly associated with PD-L1 blockade response in the IC0 and IC1 phenotypes (two-tailed t-test p = 0.021 and 0.0013).

ICB therapies have been extensively used to treat various cancers, including LUAD, and have achieved durable responses in some patient populations. However, the intrinsic cancer heterogeneity, such as the cytokine signature, largely determines the therapy outcome. Many studies have reported the disturbance of TGF-β in the ICB working process 18. For example, TGF-β blocks the effects of anti-PD-L1 therapy in urothelial cancer by facilitating exhaustion of T cells 19. The functional enrichment analysis (mentioned above) indicated that CCL20 expression is associated with immune-related pathways in LUAD, therefore we further investigated the interplay between CCL20 and TGF-β in immunotherapy using a large RNA-seq dataset (n=348) of urothelial cancer patients treated with the anti-PD-L1 agent (atezolizumab) 19. Responders are those who achieved complete response (CR) or partial response (PR), and non-responders are defined as stable (SD) or progressive disease (PD). Patients with high CCL20 expression had poor survival in metastatic urothelial cancer (Figure 6A). Although the p-value between the CCL20-high and CCL20-low subgroup while comparing the percentage of SD/PD or CR/PR was not significant, to some extent, it suggested patients with high CCL20 expression were likely to have adverse responses to atezolizumab (Figure 6B). Meanwhile, TGF-β expression was positively correlated with poor survival and non-response to atezolizumab (p=0.0018, Figure 6C-D). We divided the cohorts into four subgroups by enrolling the expression levels of CCL20 and TGF-β concurrently and found that the subgroup patients with high CCL20 and high TGF-β expression had the poorest prognosis. Moreover, low CCL20 expression blocked the detrimental effects of high TGF-β on survival. The subgroup with high CCL20 and low TGF-β expression has a similar prognosis to the CCL20-low TGF-β-high subgroup (Figure 6E). There were significant differences in the atezolizumab response among the four subgroups, and when comparing the first bar and the third bar in Figure 6F, we found that the downregulation of CCL20 further blocked the poor effects of TGF-β on the atezolizumab efficacy. Otherwise, we found that TGF-β significantly affected the ability of CCL20 to suppress the response to immunotherapy (p=0.0133 and p=0.0013, Figure 6F). These findings indicated the synergistic adverse effects of CCL20 and TGF-β on prognosis and ICB therapy efficacy.

The immune states of the tumor microenvironment drastically contribute to ICB treatment outcomes. Based on the IHC score of PD-L1 expression on immune cells, tumors can be classified into IC0 (<1% of tumor-infiltrating immune cells), IC1 (1% to <5%), and IC2/3 (≥5%). High PD-L1 expression on tumor cells and tumor-infiltrating immune cells is a predictor for improving atezolizumab response in NSCLC 20, and atezolizumab also exhibited a durable activity in locally advanced and metastatic urothelial carcinoma patients with higher PD-L1 expression on immune cells 21. To disclose the impacts of CCL20 and TGF-β on ICB therapy in different immune tumor microenvironments, we divided the urothelial cancer cohort into 3 groups according to the IHC score of PD-L1 expression on immune cells and compared the expression level of CCL20 and TGF-β in CR/PR and SD/PD subgroups. We found that high CCL20 expression was significantly associated with worse therapeutic reactivity in the IC1 subgroup (p=0.016, Figure 6G), and high TGF-β was correlated with the poor response to atezolizumab in the IC0 and IC1 subgroup (p=0.021 and p=0.0013, Figure 6H). Taken together, we validated that CCL20 and TGF-β have a synergistic inhibitory effect on ICB working efficiency in urothelial cancer.

## Discussion

In this study, we thoroughly explored the prognostic ability and biological characteristics of CCL20 in LUAD. The study is the first systemic study that discloses the role of CCL20 in LUAD progression and cancer immunotherapy based on the maximum sample size. Data analysis demonstrated that TNF, EMT, and immune-inflammatory pathways are activated in LUAD patients with high CCL20 expression. *In vitro* experiment validated that CCL20 knockdown repressed the EMT process and enhanced cell apoptosis of LUAD cancer cell lines. *In vivo* experiment showed that tumor growth was remarkably repressed after CCL20 knockdown. In addition, we revealed that high CCL20 and TGF-β expression collectively led to poor survival of patients and impaired anti-PD-L1 therapy efficacy based on a urothelial cancer dataset.

EMT is a critical stage of cancer metastasis. Previous studies reveal that CCL20 secreted by tumor-associated macrophages can promote cancer cells' EMT and migration ability via AKT activation in renal cell carcinoma 22. Similar effects of CCL20 on cancer cells are also displayed in pancreatic cancer 23. Considering the combination of CCL20 and TGF-β is associated with patients' worse prognoses and TGF-β plays a driver role in the cancer EMT 17, we raise that EMT is the predominant mechanism by which CCL20 promotes LUAD.

Intriguingly, we uncover that CCL20 is implicated in regulating programmed cell deaths for the first time. *In vitro* experiment validated that CCL20 knockdown promotes cell apoptosis in A549 and H358 cell lines. Bioinformatical analysis showed the association between other programmed cell deaths and CCL20, including autophagy, ferroptosis, and pyroptosis. Their roles in cancer are complicated 24. The effect of autophagy on the cancer cell is dependent on cellular contents and cancer types. Sometimes autophagy has a repressive impact on cancer development by inducing cell death. Oppositely, autophagy can also provide nutrition to support cancer cell survival. Studies indicated that autophagy promotes LUAD cell proliferation and tumor xenograft growth 25. In our study, CCL20 is negatively correlated with autophagy pathways, and the underlying molecular interplay needs more exploration.

Ferroptosis and pyroptosis are two novel cell death manners. Numerous studies have identified their anti-tumor activity in various cancers. Iron-dependent lipid peroxidation and ROS accretion in cells trigger ferroptosis. Taking advantage of cancer cells' high iron requirement for fast proliferation, modulating the ferroptosis process to target cancer without harming normal tissues becomes possible. The development of agents which selectively induce ferroptosis in cancer cells is a promising therapy for cancer treatment. In our study, the expression of CCL20 has negatively correlated with transferrin receptor ES and positively correlated with NADPH oxidase ES. We think that the oncogenic ability of CCL20 is at least partially dependent on repressing ferroptosis. Notably, some studies found that the EMT process and TGF-β1 promote ferroptosis in several cancer types 26, 27, and lipid peroxidation can cause EMT and ferroptosis in A549 cells synchronously 28. The specific mechanisms between CCL20, EMT, and ferroptosis need more study.

Pyroptosis is mainly induced under inflammatory states like a microbial infection. NLRP3 is a critical component of the pyroptosis signaling pathway, which senses an extensive range of stimuli and initiates subsequent signaling transduction 29. After pyroptosis, cells release IL-18 and IL-1 to trigger the following inflammatory reactions. Chemotherapy drugs like cisplatin attenuate lung cancer growth by triggering pyroptosis 30, and pyroptosis can inhibit the EMT process of cancer cells 31. CCL20 expression is positively correlated with NLRP3, IL-18, and IL-1 activity in our study. Given the CCL20/CCR6 axis plays a critical role in inflammatory diseases 32, we speculate that high CCL20 expression may be associated with the locally active inflammatory state of the tumor, which enhances the pyroptosis marker expression.

CCL20/CCR6 axis works on cancer cells in autocrine and paracrine manners. The axis not only regulates the malignant behaviors of cancer cells, but also shapes the tumor immune microenvironment by remodeling immune cell infiltration, and the effects are conducive to tumor development in most cases. For example, colorectal cancer cells highly secrete CCL20 to attract regulatory T cells (Tregs) into the tumor microenvironment, which will enhance the chemoresistance of cancer 33. IL-17-producing T cells can also be recruited by CCL20 into TME and regulate the response to ICB therapy 34, 35. However, sometimes CCL20 can promote anti-tumor immunity via the reconstruction of TIME. CCL20 produced in tumor tissues attracts circulating dendritic cells to TME, and more dendritic cells promote T cells' activation and killing ability, leading to tumor regression 36. Similarly, CCL20 injected into patients also increases the number of dendritic cells in gastric carcinoma 37. The role of CCL20 in cancer is dependent on the specific cancer context.

TGF-β is a well-described adverse factor for ICBs therapy. The efficacy of the bifunctional fusion protein targeting both TGF-β and PD-L1, Bintrafusp alfa, has been seen in clinical trials of several cancers 38-40. We showed that EMT-mediated by TGF-β is the core pathway through which CCL20 promotes LUAD, and both CCL20 and TGF-β expression are associated with poor survival in LUAD. Furthermore, we validated that high CCL20 and TGF-β expression contributed to the poor response of patients to atezolizumab in urothelial cancer cohorts. Downregulating CCL20 expression can restrain the inhibitory effects of TGF-β on ICB therapy. So, it is convincible to consider that CCL20 is a promising therapeutic target for enhancing immunotherapy efficacy.

Our study shed light on the tumor-promoting role of CCL20 in LUAD, but more experimental evidence is needed to validate the reliability of conclusions given by bioinformatical analysis and to elucidate related molecular pathways, including the mechanisms by which CCL20 regulates programmed cell death and EMT. Moreover, to explain the synergetic effects of CCL20 and TGF-β on the patients' poor response to ICB therapy, the specific molecular mechanisms between them need more investigation. In addition, the generality of the oncogenic impact of CCL20 in other cancer types warrants further validation. Future studies should focus on how to take advantage of chemokines for cancer treatment.

In conclusion, CCL20 is a novel prognostic predictor of LUAD. Downregulated expression of CCL20 can repress EMT signaling pathways in LUAD cells and restrain tumor growth. Moreover, high CCL20 expression can impair the efficacy of ICB therapy. Taken together, we highlight that CCL20 is a prognostic marker and a promising target for the improvement of immunotherapy.

## Materials and Methods

### Dataset source and preprocessing

We enrolled four independent public LUAD cohorts, including a total of 1544 cases. 468 LUAD cases with raw RNA-sequencing (RNA-seq) and clinical annotation were acquired from The Cancer Genome Atlas (TCGA) website (https://portal.gdc.cancer.gov/). The other three independent cohorts with a large population of LUAD patients with microarray data and clinical characteristics including 226 cases in GSE31210, 442 cases in GSE68465, and 398 cases in GSE72094 were downloaded from Gene Expression Omnibus (GEO) datasets (http://www.ncbi.nlm.nih.gov/geo). R version 4.0.5 software was used to normalize and process the data. The RNA-seq and clinical data of the urothelial cancer cohort are cited from the literature 19.

### Survival analysis and meta-analysis

The correlation between CCL20 expression levels alone or plus TGF-β expression levels and LUAD patients' clinical outcomes were analyzed in TCGA and GEO datasets using the R package “survival”. Meta-analysis was performed using the R package “meta”.

### Functional enrichment analysis

The differential expression gene (DEG) analysis of LUAD cases with high and low CCL20 expression levels was conducted on the TCGA dataset. A volcano plot showed the fold change and P values of DEGs between high CCL20 expression and low CCL20 expression groups. A heat map showed the TNF, EMT, and cancer promoting-inflammatory response (CP-IR)-related genes expression profile of CCL20 high and low expressed groups. The Gene Ontology (GO) and Kyoto Encyclopaedia of Genes and Genomes (KEGG) pathway analyses of the identified genes were performed using R packages “clusterProfiler”, “org.Hs.eg.db”, “enrichplot”, and “ggplot2”.

### GSVA and GSEA analysis

Gene set variation analysis (GSVA) was implemented to evaluate the association between CCL20 and different cell death pathways for each sample in TCGA and GEO datasets. The GSVA score presented the degree of absolute enrichment of a gene set in samples. Pearson's correlation analysis was performed to investigate the relationship between CCL20 and three tumorigenesis pathways. GSEA hallmark term analysis and GO term analysis were conducted for high and low CCL20 expression groups using GSEA software (V.4.1.0) to identify related signaling pathways.

### Cell lines and cell culture

The lung adenocarcinoma cell lines A549, and H358 were obtained from American Type Culture Collection (ATCC). Cells were cultured in medium RPMI 1640 (Gibco, California, USA) supplemented with 10% fetal bovine serum (FBS, Gibco), 100 U/ml penicillin, and 100 μg/ml streptomycin at 37℃, 5% CO_2_.

### Cell transfection

According to the manufacturer's protocol, A549 and H358 cells were transfected with 50 nM double-stranded siRNA oligonucleotides twice, which was synthesized by Generay Biotech, Shanghai, China. The CCL20 siRNA sequences were as follows: 5'-3' GGUUUAGUGCAAAGUAUAATT, 3'-5' UUAUACUUUGCACUAAACCTT. Lipofectamine™ 3000 Transfection Reagent was purchased from Thermofisher (Massachusetts, USA).

### Cell proliferation and cycle assay

Cell proliferation was assessed by Cell Counting Kit-8 (CCK-8). 5×10^3^ A549 cells were plated in 96 well plates (100μL/well) and incubated in the incubator (37℃, 5%CO_2_) after adding 10μL CCK-8 to each well for 1-4 hours. The OD values were measured at 450nm with a microplate reader. The same method was applied for H358 cells.

Propidium iodide (PI) stain was used for cell cycle assay. Counting 5×10^5^ cells were harvested for trypsinization, washed with cold PBS, and fixed with absolute ethanol on ice. After discarding the supernatant and washed with PBS one time, cells were treated with RNase A (0.5mg/mL) for 30 min at 37°C. Cell suspensions were stained with PI (50 μg/mL) in the dark at 4°C for 40 min. Stain detection was performed using a BD Flow cytometer (BD Pharmingen), and the cell cycle was analyzed using Modfit software.

### Cell apoptosis analysis

For the apoptosis assay, cells were treated with 30μM cisplatin for 24h, and were washed by cold PBS two times followed by cell suspension was prepared with trypsinization and centrifugation. The PBS liquid and medium were all collected to stain with FITC Annexin V/propidium iodide (PI) (BD Biosciences) and examined by NovoCyte Flow Cytometer (ACEA Biosciences) and data was analyzed by FlowJo V10.

### Protein extraction and western blot

For protein extraction, the treated cells were harvested and lysed with RIPA lysis buffer (Servicebio, Wuhan, China) containing proteinase inhibitor cocktail (APPLYGEN, Beijing, China) on ice for 15 min, and the supernatants were stored at -80℃ after the centrifugation of lysis liquid. The concentrations of total proteins were measured by a BCA protein assay (Pierce, ThermoFisher Scientific), and equal amounts of proteins were used for western blot to quantify target proteins.

Within western blot, proteins were separated by SDS-PAGE gel electrophoresis and transferred onto PVDF membranes (Millipore). Following blocked with 5% BSA solution for 1 hour, the membranes were incubated at 4 °C overnight with primary antibodies to EMT-related proteins (ZO-1(CST 8193), N-Cadherin(CST 13116), E-Cadherin(CST 3195), β-Catenin(CST 8480), Vimentin(CST 5741)) and GAPDH(CST 5174). Then membranes were incubated with HRP-conjugated anti-rabbit secondary antibody for 2 hours at room temperature, and the protein bands were detected with chemiluminescence detection kit (Immobilon™ Western Chemiluminescent HRP Substrate) and imager.

### Animal studies

Within the establishment of the xenograft tumor model, 5 × 10^6^cells of A549 cells transient transfected with either control or CCL20 siRNA were subcutaneously implanted into the right axillar of 4-5 week-old BALB/c nude mice (n= 6 per group). Starting from day 7, tumor size was monitored every three days for a total of 28 days. Finally, mice were euthanized for tumor excision. The animal experiment was performed conducted following a protocol approved by the Institutional Animal Care and Use Committee of the Cancer Hospital, Chinese Academy of Medical Sciences.

### Statistical analysis

R version 4.0.5 and GraphPad Prism 8.4.3 were used for data analysis. The significance of the difference in prognostic analyzes was evaluated via log-rank test. The hazard ratios were calculated by the univariate Cox regression model. Correlations coefficients between CCL20 and TNF, EMT, and CP-IR gene sets were computed by Spearman correlation analysis. Significances of difference between two groups were evaluated using a two-tailed unpaired Student's t-test. All data were represented as mean ± SEM. Statistical P values <0.05 were considered statistically significant.

## Supplementary Material

Supplementary figures.

Supplementary table.

## Figures and Tables

**Table 1:** Univariable and multivariable Cox regression analysis of CCL20 in TCGA dataset

	Univariable analysis			Multivariable analysis		
Characteristics	HR	95%CI	*P* Value	HR	95%CI	*P* Value
Age						
≤65 or >65	1.287	0.942-1.759	0.113			
Gender						
Female or Male	0.826	0.606-1.127	0.229			
Smoking history						
Yes or No	0.971	0.624-1.513	0.897			
TNM stage						
I, II, III or V	1.586	1.37-1.835	<0.001	1.293	1.03-1.624	0.027
T stage						
1, 2, 3 or 4	1.594	1.314-1.934	<0.001	1.338	1.084-1.651	0.007
N stage						
0, 1, 2 or 3	1.626	1.362-1.941	<0.001	1.19	0.931-1.521	0.165
CCL20						
High or low	1.003	1.001-1.005	0.013	1.003	1-1.005	0.018

**Table 2:** Clinical characteristics of lung adenocarcinoma from multiple cohorts

Characteristics	TCGA cohortN=477	GSE31210N=226	GSE72094N=398	GSE68465N=443
Age, year	66(59-72)	61(55-65)	70(64-77)	65(58-72)
Gender				
Male	217	105	176	223
Female	260	121	222	220
Stage				
I	258	168	254	/
II	115	58	67	/
III	78	0	57	/
IV	25	0	15	/
NA	1	0	5	/
Chemotherapy				
Yes	55	/	/	89
No	74	/	/	341
NA	348	/	/	13
Radiotherapy				
Yes	59	/	/	65
No	73	/	/	364
NA	345	/	/	14
Status				
Alive	296	191	285	207
Death	181	35	113	236
RFS				
No	205	162	/	157
Yes	233	64	/	205
NA	39	0	/	81

## Abbreviations

LUAD: Lung adenocarcinoma
OS: Overall survival
RFS: Relapse-free survival
EMT: Epithelial-mesenchymal transition
TME: Tumor microenvironment
ICB: Immune checkpoint blockade
TCGA: The Cancer Genome Atlas
GEO: Gene Expression Omnibus
DEG: Differential expression gene
CP-IR: Cancer promoting-inflammatory response
GO: Gene Ontology
KEGG: Kyoto Encyclopaedia of Genes and Genomes
GSVA: Gene set variation analysis
CR: Complete response
PR: Partial response
SD: Stable disease
PD: Progressive disease
