# Supplementary material for: CCL20 promotes lung adenocarcinoma progression by driving epithelial-mesenchymal transition
Source: Int J Biol Sci. 2022 Jun 27;18(11):4275–88. doi: 10.7150/ijbs.73275 (PMC9295072; doi:10.7150/ijbs.73275)
Supplement: Supplementary file 1 — Supplementary figures. [file ijbsv18p4275s1.pdf]

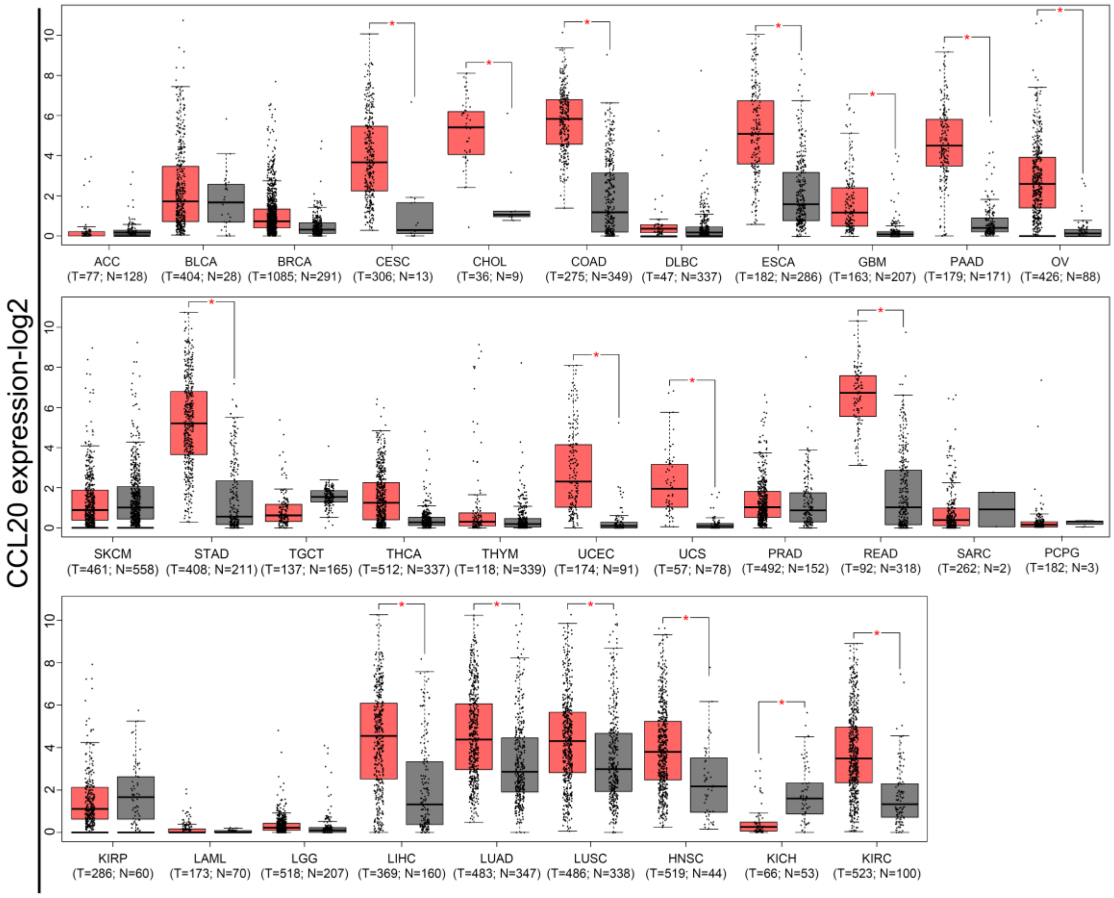

Figure S1. Landscape of CCL20 expression in pan-cancer.

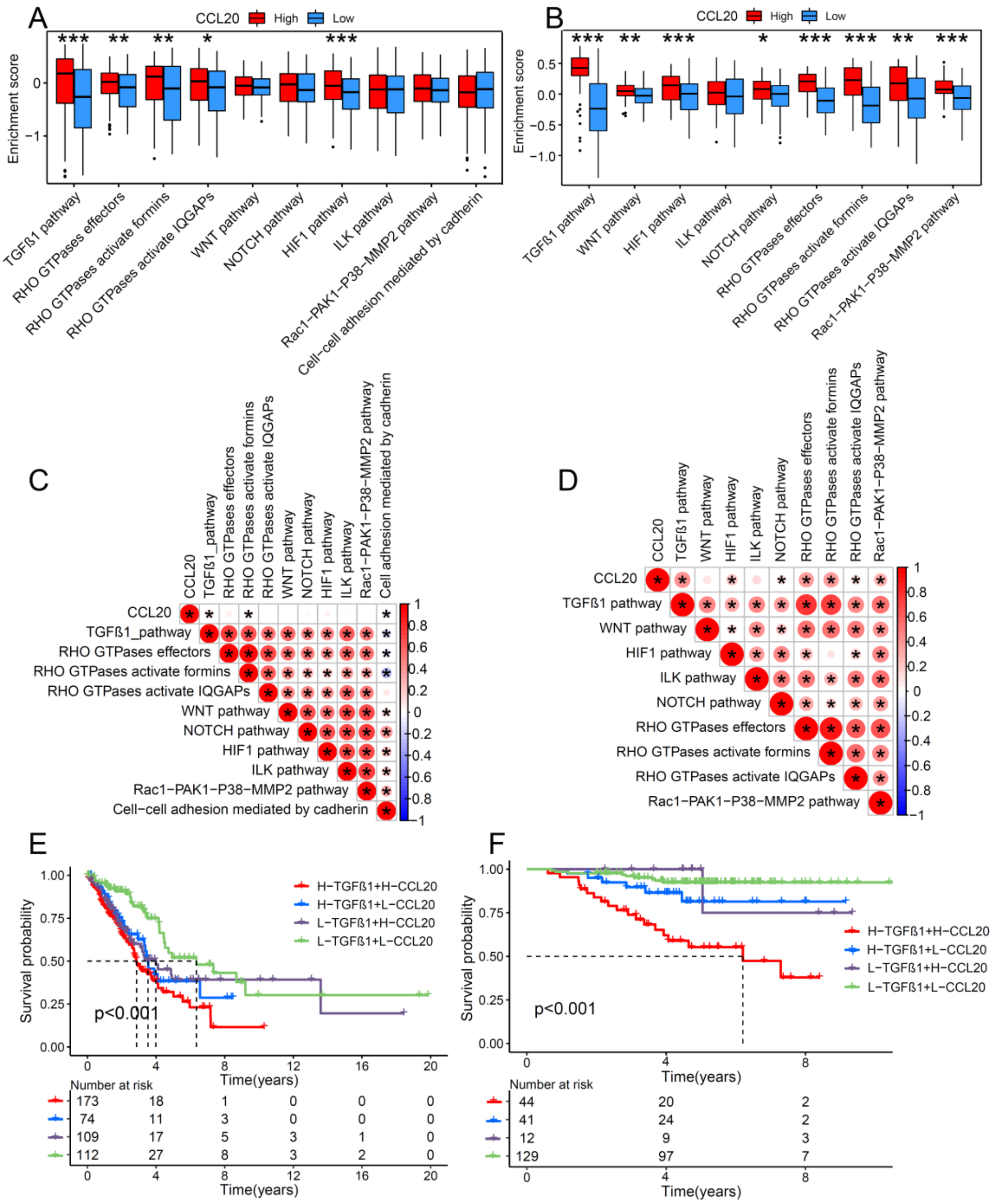

Figure S2. The relationship between CCL20 and EMT-related pathways. Differences in EMT-related pathways between high CCL20 and low CCL20 groups in TCGA cohort (A) and GSE31210 cohort (B). Correlation analysis of CCL20 and EMT-related pathways in TCGA cohort (C) and GSE31210 cohort (D). OS analysis for subgroup patients stratified by both CCL20 and TGF-β in TCGA cohort (E) and GSE31210 cohort (F)

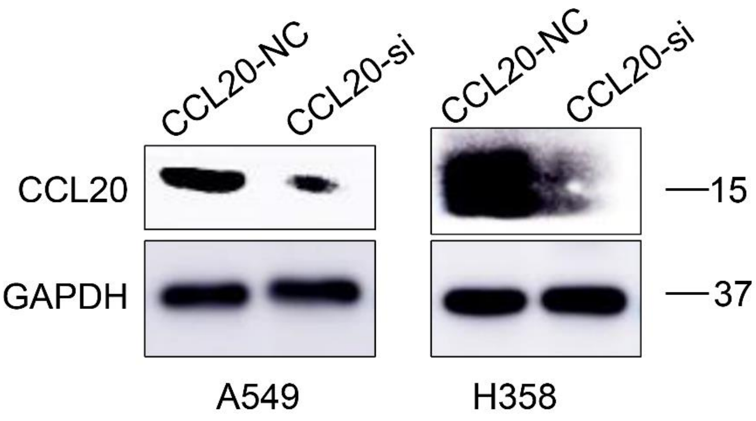

Figure S3. The knockdown efficiency of CCL20 in A549 and H358 cell lines was examined by western blot. GAPDH was served as control.
